# The effect of polygenic liability to mental disorders on COVID-19 outcomes in people with depression: the mediating role of anxiety

**DOI:** 10.1017/S0033291724001983

**Published:** 2024-11

**Authors:** Anna Monistrol-Mula, Mireia Felez-Nobrega, Enda M. Byrne, Penelope A. Lind, Ian B. Hickie, Nicholas G. Martin, Sarah E. Medland, Lucía Colodro-Conde, Brittany L. Mitchell

**Affiliations:** 1 Group of Epidemiology of Psychiatric disorders and Ageing, Sant Joan de Déu Research Institute, Sant Boi de Llobregat, Barcelona, Spain; 2 Centre for Biomedical Research on Mental Health (CIBERSAM), Madrid, Spain; 3 Department of Medicine, University of Barcelona, Barcelona, Spain; 4 Child Health Research Centre, The University of Queensland, Brisbane, Australia; 5 Mental Health and Neuroscience Research Program, QIMR Berghofer Medical Research Institute, Brisbane, QLD, Australia; 6 School of Biomedical Sciences, Queensland University of Technology, Brisbane, Australia; 7 School of Biomedical Sciences, University of Queensland, Brisbane, Australia; 8 Brain and Mind Centre, The University of Sydney, Sydney, NSW, Australia; 9 School of Psychology, The University of Queensland, Brisbane, QLD, Australia; 10 School of Psychology and Counselling, Queensland University of Technology, Brisbane, Australia

**Keywords:** genetic vulnerability, polygenic risk scores (PRS), depression, COVID-19 outcomes, anxiety symptoms, COVID-19 burnout, mediation analysis, SARS-CoV-2 infection, resilience

## Abstract

**Background:**

Genetic vulnerability to mental disorders has been associated with coronavirus disease-19 (COVID-19) outcomes. We explored whether polygenic risk scores (PRSs) for several mental disorders predicted poorer clinical and psychological COVID-19 outcomes in people with pre-existing depression.

**Methods:**

Data from three assessments of the Australian Genetics of Depression Study (*N* = 4405; 52.2 years ± 14.9; 76.2% females) were analyzed. Outcomes included COVID-19 clinical outcomes (severe acute respiratory syndrome coronavirus 2 [SARS-CoV-2] infection and long COVID, noting the low incidence of COVID-19 cases in Australia at that time) and COVID-19 psychological outcomes (COVID-related stress and COVID-19 burnout). Predictors included PRS for depression, bipolar disorder, schizophrenia, and anxiety. The associations between these PRSs and the outcomes were assessed with adjusted linear/logistic/multinomial regressions. Mediation (*N* = 4338) and moderation (*N* = 3326) analyses were performed to explore the potential influence of anxiety symptoms and resilience on the identified associations between the PRSs and COVID-19 psychological outcomes.

**Results:**

None of the selected PRS predicted SARS-CoV-2 infection or long COVID. In contrast, the depression PRS predicted higher levels of COVID-19 burnout. Anxiety symptoms fully mediated the association between the depression PRS and COVID-19 burnout. Resilience did not moderate this association.

**Conclusions:**

A higher genetic risk for depression predicted higher COVID-19 burnout and this association was fully mediated by anxiety symptoms. Interventions targeting anxiety symptoms may be effective in mitigating the psychological effects of a pandemic among people with depression.

## Introduction

The emergence of the coronavirus disease-19 (COVID-19), caused by severe acute respiratory syndrome coronavirus 2 (SARS-CoV-2), became a global health crisis, first, due to its rapid spread and high mortality rates, and second, due to the persistent form of COVID-19, known as long COVID, where symptoms last for months after infection (World Health Organization, 2022). Similar prolonged syndromes with physical, cognitive, and affective symptoms have been reported and followed longitudinally after other severe infections (Hickie et al., 2006). A range of concurrent mood and individual psychological traits appear to predict such prolonged illness experiences (Cvejic et al., 2019). Among these, depression, the most common mental health disorder and the largest contributor to global disability (König, König, & Konnopka, 2019), is of particular concern. Results from a recent meta-analysis suggest that individuals with depression present increased risks of severe COVID-19 and mortality than the general population (Molero et al., 2023). Moreover, this population has been reported to have an increased risk of long COVID (Wang et al., 2022), making them particularly vulnerable to COVID-19.

Prior studies suggest a potential role of genetic factors underlying the phenotypic association between mental disorders, including depression and infections (Nudel et al., 2019). These studies used polygenic risk scores (PRSs) to measure cumulative genetic risk for mental disorders. PRSs can have small effects since they explain only part of the genetic aspect of a condition, but they may still hold significant clinical utility. However, evidence on whether a genetic predisposition to mental disorders is associated with COVID-19 risk or its long-term consequences is inconsistent. For instance, one study of over 140 000 adults (50+ years), found that higher genetic predisposition to depression, anxiety, and substance use disorder, but not to psychotic disorders, increased the risk of SARS-CoV-2 infection and severe COVID-19 (W. Chen et al., 2022a). Conversely, another study of 15 000 participants found that genetic risk for schizophrenia, but not depression or bipolar disorder, predicted severe COVID-19 (Alemany-Navarro et al., 2023). However, none of these studies have focused on individuals with a mental health diagnosis.

Individuals with depression are especially susceptible to pandemic stressors, such as disrupted access to mental health services and reduced social networks, thereby increasing their risk of relapse or worsening of existing mental conditions (Yao, Chen, & Xu, 2020). Indeed, they experienced higher levels of COVID-related stress, burnout, and mental health symptoms compared to the general population (Asmundson et al., 2020; Pan et al., 2021). The psychological impact of the COVID-19 pandemic might also vary depending on the genetic predisposition to depression and other mental disorders. One study found that a PRS for a general psychopathology factor based on the aggregation of 12 PRSs for mental disorders predicted being assigned to an acute dysfunction group (those showing an increase in mental health symptoms during lockdown but a decrease in symptoms once lockdown ended) rather than a resilient group (those not presenting alterations in mental health symptoms during the COVID-19 pandemic) (Ahrens et al., 2022). Nevertheless, to the best of our knowledge, no study has yet assessed if genetic predisposition to mental disorders predicts COVID-related stress and burnout in individuals with depression.

Anxiety symptoms are common in individuals with depression (Kessler et al., 2015) and can worsen the psychological toll of the pandemic, leading to increased worry, fear, and uncertainty. Indeed, anxiety symptoms have been linked to increased COVID-related stress (Monistrol-Mula et al., 2022). Conversely, individuals with higher resilience levels are better equipped to cope with pandemic challenges, experiencing less stress and burnout associated with the COVID-19 pandemic (Armstrong et al., 2022) and prior viral epidemics (Bonanno et al., 2008).

The COVID-19 pandemic is a universal environmental stressor. This study aimed to investigate in a cohort with a history of depression whether: (1) PRSs for depression, bipolar disorder, schizophrenia, and anxiety predict susceptibility to COVID-19 disease outcomes (infection and long COVID) and COVID-19 psychological outcomes (COVID-related stress and burnout); (2) anxiety symptoms mediate the relationship between a genetic predisposition to these disorders and psychological outcomes; and (3) resilience moderates the association between genetic predisposition to the mental disorders of interest and COVID-19 psychological outcomes. These findings could inform public health policies to protect vulnerable populations and promote mental well-being during the ongoing pandemic and future epidemics.

## Methods

### Australian Genetics of Depression Study

The Australian Genetics of Depression Study (AGDS) aims to identify genetic risk factors associated with clinical depression and treatment response. Full details regarding recruitment, sample collection, and measures are described elsewhere (Byrne et al., 2020). In brief, the study comprises over 22 000 Australian adults (15 792 of whom have been genotyped) of European ancestry who have received treatment for clinical depression. Participants were recruited through two distinct approaches: by identifying individuals with a nationwide antidepressant prescription history over the past 4.5 years, and through a national media campaign. Participants completed an online baseline questionnaire, including a mandatory depression module, with optional modules on mental health, physical health, and lifestyle. Following the baseline questionnaire (2017), participants were invited to complete three follow-up surveys during the COVID-19 pandemic (2020, 2021, and 2022), which included questions regarding the impact of the COVID-19 pandemic on both their mental and physical health. This study analyzed data from the baseline questionnaire and COVID-19 follow-ups in 2021 and 2022. Of 22 289 baseline participants with age data, 25.6% (*n* = 5701) completed the 2022 COVID-19 survey, which included the main outcomes analyzed in this study. Of these, 87.1% (*n* = 4969) had been genotyped, and 77.3% (*n* = 4405) had no missing outcome variables. Finally, 58.3% (*n* = 3326) completed both the 2021 and 2022 COVID-19 surveys, where resilience data were collected.

### PRSs for mental disorders

Polygenic Risk Scores (PRSs) were calculated using the summary statistics from genome-wide association studies (GWASs) of depression (246 363 cases and 561 190 controls) (Howard et al., 2019), bipolar disorder (41 917 cases and 371 549 controls) (Mullins et al., 2021), schizophrenia (76 755 cases and 243 649 controls) (Trubetskoy et al., 2022), and anxiety (25 453 cases and 58 113 controls) (Purves et al., 2020). We used SBayesR v2.03 to generate the PRSs, which has been shown to outperform classic PRS calculation methods in the prediction of complex traits (Lloyd-Jones et al., 2019). SBayesR is a Bayesian method that re-scales the GWAS single-nucleotide polymorphism (SNP) effects with SNPs presumed to have an effect size of zero. For the Linkage Disequilibrium (LD) reference, we used one LD matrix based on the HapMap3 SNPs of 50 000 unrelated individuals randomly selected from the UK Biobank (Lloyd-Jones et al., 2019). The posterior SNP effects estimated by SBayesR were used to generate PRSs for each individual using the –score function in PLINK.

### Outcome variables

#### COVID-19 disease outcomes

Disease outcomes were assessed in the 2022 COVID follow-up questionnaire (completed between May and June) and included SARS-CoV-2 infection and long COVID. SARS-CoV-2 infection was based on the number of self-reported COVID-19 diagnoses. Only those infections diagnosed with a PCR, a rapid antigen test. or by a doctor were considered as positive diagnosis of COVID-19. Likewise, only those reporting never being diagnosed with COVID-19 were considered as negative cases. Participants who reported a probable diagnosis of COVID-19 (having potential symptoms but not getting tested) (*n* = 127) were excluded. One participant who reported an implausible number of infections (*n* = 18) was also excluded. A three-level variable was created for SARS-CoV-2 infection: never had COVID-19, had COVID-19 once, and had COVID-19 twice. Importantly, at the time of this study the population incidence of confirmed COVID-19 infections in Australia was low compared with many other countries. Participants were considered to have long COVID if they reported having COVID-19 at least 3 months ago and reported experiencing fatigue, shortness of breath, and/or brain fog for at least 2 months following the COVID-19 diagnosis. A dichotomous variable was created for long COVID (yes/no).

#### COVID-19 psychological outcomes

Psychological outcomes of the COVID-19 pandemic were assessed in the 2022 COVID-19 follow-up questionnaire and included COVID-related stress and COVID-19 burnout. COVID-related stress refers to the psychological and emotional strain experienced by individuals in response to the COVID-19 pandemic (Taylor, 2021). COVID-related stress was assessed with six items evaluating how much stress the following situations caused in the prior 2 weeks: you or others catching COVID-19, the impact of COVID-19 on your physical/mental health, being lonely during the pandemic, and following social distancing recommendations (some items were based on the COVID Worries domain of the CRISIS questionnaire) (Nikolaidis et al., 2021). Each item was rated on a five-level scale ranging from not at all (0) to extremely worried (4). The total score was obtained by adding all responses (0–24), where higher scores reflected higher levels of COVID-related stress.

COVID-19 burnout refers to a state of physical, emotional, and mental exhaustion experienced by people as a result of the prolonged exposure to the COVID-19 pandemic stressors (Queen & Harding, 2020). COVID-19 burnout was evaluated using an adapted version of the COVID-19 Burnout Scale (Yildirim & Solmaz, 2020) (the item *When you think about COVID-19 overall, how often do you feel ‘I've had it’?* was excluded). The resulting nine-item questionnaire assessed how frequently you experienced tiredness, disappointment, depression, hopelessness, helplessness, physical weakness or sickness, feeling trapped, worthlessness, and sleep difficulties when thinking about COVID-19. Each item was rated from never (0) to always (4), with a total score ranging from 0 to 36.

### Anxiety symptoms (mediator) and resilience (moderator)

We used the seven-item Generalized Anxiety Disorder Scale (GAD-7) in the COVID-19 follow-up to screen for anxiety symptoms, with items describing problems related to anxiety and participants responding how often they have been bothered by them, with answers ranging from 0 (not at all) to 3 (nearly every day). The total sum scores range from 0 to 21, with higher scores showing higher levels of anxiety symptoms (Spitzer, Kroenke, Williams, & Löwe, 2006). We used the six-item Brief Resilience Scale (BRS) in the 2021 COVID-19 follow-up to screen for resilience. The authors of this scale define resilience as ‘the ability to bounce back or recover from stress’ (Smith et al., 2008). Thus, the six items from the BRS assess your agreement with statements related to your ability to recover after hard times, how fast you recover from stressful events, and how you unfold through stressful situations, with responses varying from 1 (strongly disagree) to 5 (strongly agree). The item average divided by the total number of items results in scores ranging from 1 to 5, with higher scores showing higher resilience symptoms.

### Covariates

Covariates used in all statistical analysis were eight genetic principal components, sex, severity of depression history, and age from the 2022 COVID-19 survey. Severity of depression history was assessed in the baseline questionnaire and was based on the number of self-reported lifetime depressive episodes lasting at least 2 weeks (1–13+). Self-reported smoking, comorbid mental disorders (including bipolar disorder, schizophrenia, anorexia nervosa/bulimia, attention-deficit/hyperactivity disorder, autism spectrum disorder, Tourette's disorder, anxiety disorder, panic disorder, obsessive compulsive disorder, hoarding disorder, posttraumatic stress disorder, phobias, seasonal affective disorder, premenstrual dysphoric mood disorder, personality disorder, and substance use disorder) and physical diseases (including cancer, diabetes, hypertension, renal disease, lung disease, and heart disease) were also tested as covariates in our analyses, but given that these variables did not contribute to any of the regression models, they were not included in the final analyses.

### Statistical analysis

The association between each PRS (depression, bipolar disorder, schizophrenia, and anxiety) and our outcome variables was estimated using linear regression for continuous variables (COVID-related stress and COVID-19 burnout), logistic regression for the binary long COVID variable, and multinomial regression for the categorical SARS-CoV-2 infection variable. All PRSs were standardized to a normal distribution, so each unit increase corresponded to one standard deviation increase in genetic predisposition. All previously described confounders were included in each analysis. Models with all PRSs predicting each outcome were also fitted. Odds ratios (ORs), relative risk ratios (RRRs), and 95% confidence intervals (CIs) were calculated where appropriate. Sex-stratified analyses were also conducted. Differences in mean PRS among SARS-CoV-2 infection levels were assessed using analysis of variance (ANOVA). Participants were divided by PRS deciles, and results were plotted to show the log OR of COVID-related stress and COVID-19 burnout for each PRS decile relative to the lowest decile.

To understand the potential mediating role of anxiety symptoms on the identified associations between the psychiatric PRSs and COVID-19 psychological outcomes, we performed a mediation analysis. This method decomposes the full effect of a variable into direct effects, this is, the effect of psychiatric PRSs (independent variable) on COVID-19 psychological outcomes (outcome), without considering the anxiety symptoms (mediator), and indirect effects (the effect of anxiety symptoms on COVID-19 psychological outcomes due to the psychiatric PRS). We then quantified the percentage of mediation explained by anxiety symptoms on our main association through non-parametric bootstrap techniques with 5000 simulations (Alfons, Ateş, & Groenen, 2022). Bootstrap is superior to other methods to test the significance of indirect effects as it makes fewer assumptions (Alfons et al., 2022). Therefore, it is applicable in a wider variety of situations, providing generic ways to consistently build CIs for indirect effects (Alfons et al., 2022).

Finally, we tested the potential moderating effect of resilience on the identified associations by adding the interaction term between resilience and the corresponding PRS, together with the first-order interactions between covariates, in separate linear regression models predicting COVID-related stress and COVID-19 burnout where all variables had been centered.

To account for multiple testing we used matrix spectral decomposition for the correlation matrix of all the outcomes (Nyholt, 2004), and set the significance threshold to 0.013 for exploratory analysis. Statistical analyses were performed with R (version 4.2.0) and the R packages *mediation* and *lmtest* (Tingley, Yamamoto, Hirose, Keele, & Imai, 2014).

## Results

A total of 4405 participants with a history of depression were included in the current study. The sample characteristics are shown in Table 1. The mean age was 52.2 years (s.d.: 14.9) and 76.2% were female. Nearly one-quarter (22.7%) of participants reported being infected with SARS-CoV-2 once, while 0.7% had been infected twice at survey time. Approximately 3.8% reported suffering from long COVID.

**Table 1.** Demographics of the study population

Variables		*N* = 4405
Age, *mean* (s.d.)		52.2 (14.9)
Depression severity, *mean* (s.d.)		7.8 (4.8)
Anxiety symptoms, *mean* (s.d.)^a^		6.6 (5.3)
Resilience, *mean* (s.d.)^b^		2.8 (0.8)
Sex, *n* (%)	Female	3366 (76.2)
	Male	1051 (23.8)
*Outcomes*		
SARS-CoV-2 infection, *n* (%)	Never infected	3387 (76.6)
	Once	1001 (22.7)
	Twice	29 (0.7)
Long COVID-19, *n* (%)	No	4249 (96.2)
	Yes	168 (3.8)
COVID-related stress, *mean* (s.d.)		8.6 (4.8)
COVID burnout, *mean* (s.d.)		9.7 (7.7)

*Note*: The sample variable was reduced in some variables,

a *n* = 4338.

b *n* = 3326.

### PRS prediction of COVID-19 clinical outcomes

**Figure 1.**
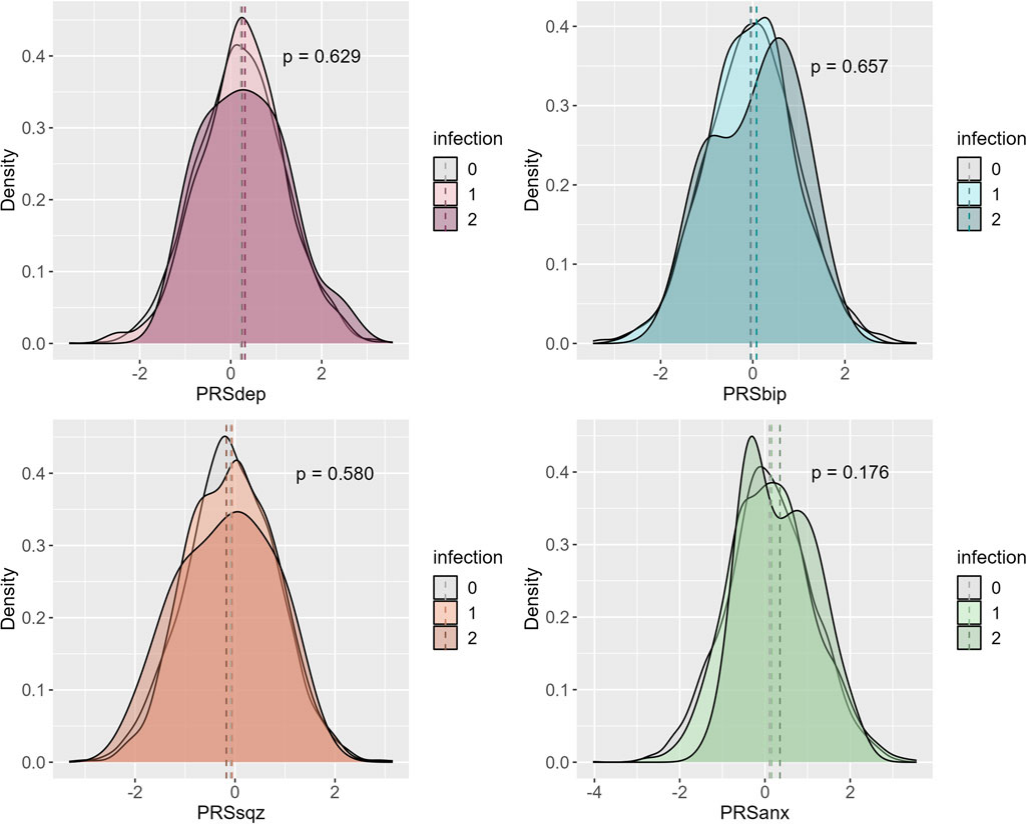
PRS prediction of SARS-CoV-2 infection. *p*-values were obtained using an ANOVA test facing PRS mean and SARS-CoV-2 infection. PRSdep, PRS for depression; PRSbip, PRS for bipolar disorder; PRSsqz, PRS for schizophrenia; PRSanx, PRS for anxiety.

Using the PRS for depression, bipolar disorder, schizophrenia, and anxiety as proxies for the genetic predisposition to the corresponding disorders, we analyzed whether a higher genetic predisposition to these mental disorders predicted our COVID-19 clinical outcomes of interest. However, we did not observe that the genetic risk for any of the included mental disorders significantly increased the risk of SARS-Co-V-2 infection (Table 2). However, a non-significant shift toward an increased genetic risk of anxiety disorder was observed among those having two COVID-19 infections (Fig. 1). Likewise, our results did not show an association between an increased genetic predisposition to the four mental disorders and long-COVID (Table 2). These results were maintained when models were fitted including all four PRSs (online Annex 1), when models were adjusted for all covariates (online Annex 2), and in sex-stratified models (online Annex 3).

**Table 2.** Results of regression analysis for the clinical and psychological COVID-19 outcomes (*N* = 4405)

Outcome		PRS depression			PRS bipolar disorder			PRS schizophrenia			PRS anxiety		
		RRR (95% CI)	*p*		RRR (95% CI)	*p*		RRR (95% CI)	*p*		RRR (95% CI)	*p*	
SARS-CoV-2 infection	Never	Ref			Ref			Ref			Ref		
	Once	1.04 (0.96–1.13)	0.325		0.95 (0.88–1.03)	0.184		1.03 (0.95–1.13)	0.440		1.06 (0.98–1.14)	0.163	
	Twice	1.21 (0.81–1.81)	0.360		1.13 (0.73–1.67)	0.519		0.92 (0.60–1.40)	0.702		1.28 (0.87–1.87)	0.213	
		OR (95% CI)	*p*		OR (95% CI)	*p*		OR (95% CI)	*p*		OR (95% CI)	*p*	
Long COVID	No	Ref			Ref			Ref			Ref		
	Yes	0.97 (0.82–1.15)	0.713		1.06 (0.90–1.25)	0.505		0.96 (0.80–1.15)	0.650		1.02 (0.87–1.21)	0.757	
Outcomes		*β* (s.e.)	*p*	Adj. *R*^2^	*β* (s.e.)	*p*	Adj. *R*^2^	*β* (s.e.)	*p*	Adj. *R*^2^	*β* (s.e.)	*p*	Adj. *R*^2^
COVID-related stress		0.18 (0.08)	0.027	0.024	−0.07 (0.08)	0.352	0.023	−0.12 (0.08)	0.160	0.023	0.03 (0.08)	0.692	0.023
COVID-19 burnout		0.36 (0.12)	**0.003**	0.089	−0.24 (0.12)	0.046	0.088	−0.17 (0.13)	0.190	0.088	0.12 (0.11)	0.333	0.087

RRRs with 95% CI and *p*-values are displayed for categorical variables. ORs with 95% CIs and *p*-values are displayed for bivariate variables. Estimates (*β*) with standard errors (s.e.) and *p*-values are displayed for continuous variables. Adjusted *R*^2^ are displayed for linear models. Analysis were made separately for each outcome and PRS, and adjusted for age, sex, severity of depression, and eight genetic ancestry principal components. Bold *p*-values show significant associations (corrected significance threshold *p* < 0.013).

### PRS prediction of COVID-19 psychological outcomes

**Figure 2.**
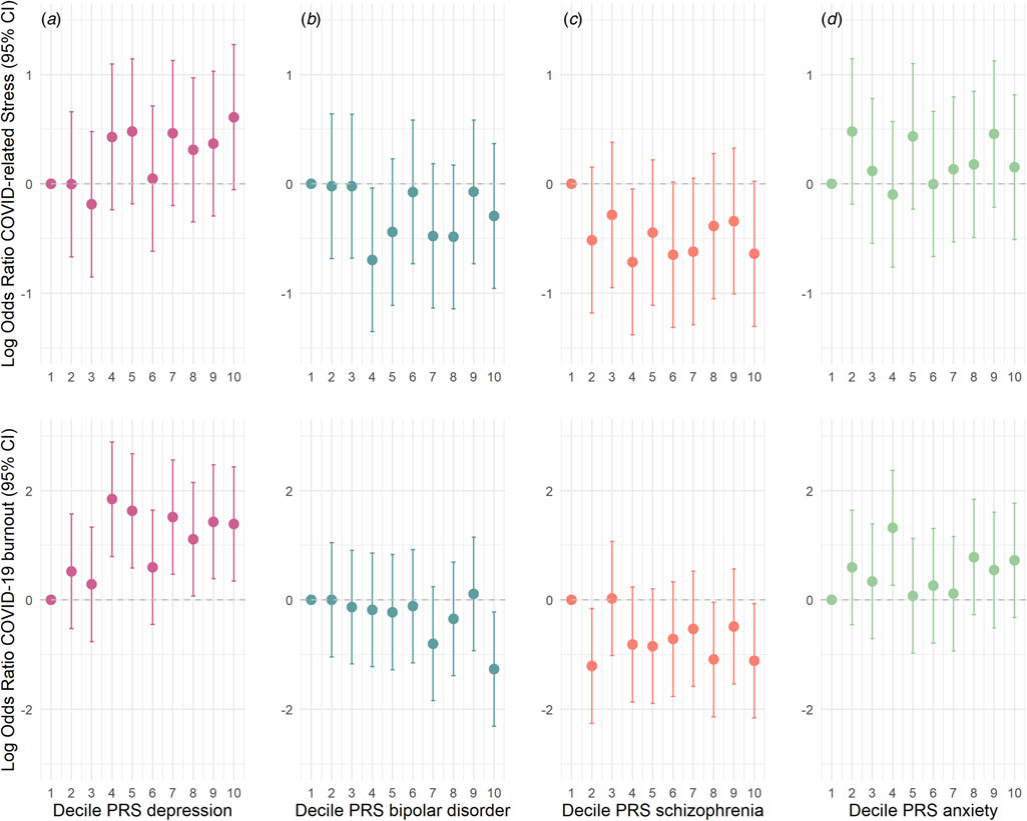
Log OR of COVID-related stress and COVID-19 burnout within each PRS decile for depression (*a*), bipolar disorder (*b*), schizophrenia (*c*), and anxiety (*d*) relative to those in the lowest decile in the AGDS.

We also analyzed whether a higher genetic predisposition to depression, bipolar disorder, schizophrenia, and anxiety predicted the COVID-19 psychological outcomes of interest. A higher genetic risk of depression predicted higher COVID-related stress and COVID-19 burnout, although the former did not survive multiple testing correction (Table 2). Individuals in the top 10% of genetic risk for depression were 1.87 (95% CI 0.96–3.63) times more likely to report higher COVID-related stress, and 4.17 (95% CI 1.47–11.86) times more likely to report higher COVID-19 burnout than individuals in the lowest 10% of genetic risk (Fig. 2). A higher genetic predisposition to bipolar disorder was nominally associated with lower COVID-19 burnout. Individuals in the top 10% of genetic risk for bipolar disorder were 0.27 (95% CI 0.09–0.76) times less likely to report higher COVID-19 burnout than individuals in the lowest 10% of genetic risk (Fig. 2). A genetic predisposition to schizophrenia and anxiety did not predict either psychological outcome (Table 2). These results were also maintained when all PRSs were included in the same model (see online Annex 1), and when models were adjusted for all covariates (see online Annex 2). When stratifying by sex, the observed associations were maintained in females, although only nominally, but not in males (online Annex 3).

### Mediation and moderation analysis

**Figure 3.**
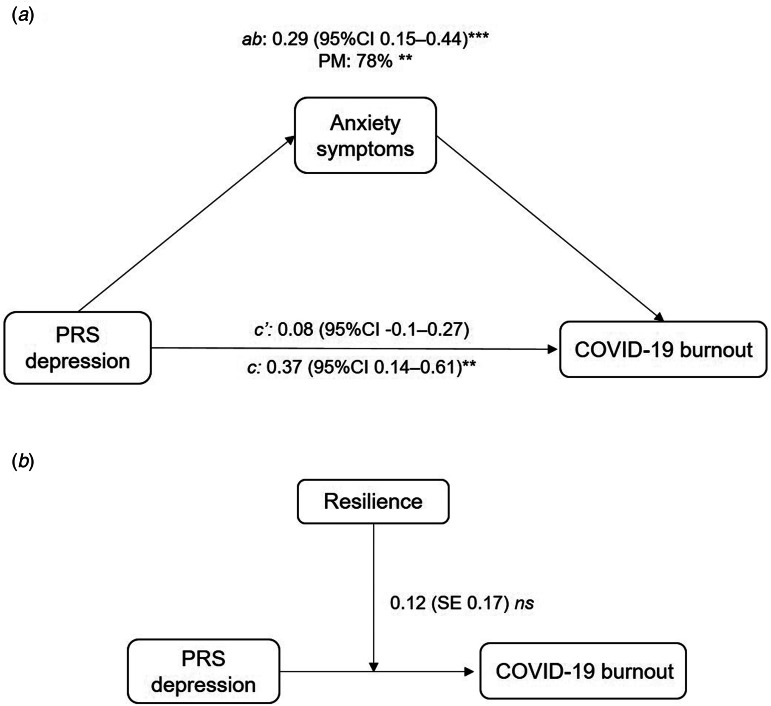
(*a*) Unstandardized coefficients and CIs for the mediation model. The ab path coefficient represents the mediation effect of anxiety symptoms on the association between PRS depression and COVID-19 burnout. The c path coefficient represents the total effect of the PRS for depression on COVID-19 burnout. The c′ coefficient represents the direct effect of the PRS for depression on COVID-19 burnout. (*b*) Standardized coefficients and standard error for the moderation effect of resilience on the association between PRS for depression and COVID-19 burnout. CI, confidence interval; s.e., standard error; PM, proportion mediated; ns, non-significant. *p < 0.05, **p < 0.01, ***p < 0.001.

We analyzed the potential mediator role of anxiety symptoms on the significant association between the PRS for depression and COVID-19 burnout (*N* = 4338). We found that anxiety symptoms significantly mediated the association, with a proportion of mediation of 78.0% (*p* *=* 0.003). Once the model included anxiety symptoms as the mediator, the direct effect of the genetic risk on COVID-19 burnout disappeared (full mediation) (Fig. 3a). Lastly, we analyzed whether resilience moderated the association between PRS for depression and COVID-19 burnout (*N* = 3326). While resilience predicted lower COVID-19 burnout, it did not significantly moderate the association between PRS for depression and COVID-19 burnout (Fig. 3b and online Annex 4).

## Discussion

We examined whether genetic risk for depression, bipolar disorder, anxiety, and schizophrenia predicted COVID-19 disease and psychological outcomes in 4405 AGDS participants who had a lifetime history of depression.

The genetic predisposition to these mental disorders did not significantly predict SARS-CoV-2, although a non-significant shift toward an increased genetic risk for anxiety was observed among those reported having had two SARS-CoV-2 infections. This is contrary to prior studies conducted in UK and Spain that have reported an association between a higher genetic risk for depression, anxiety (W. Chen et al., 2022a), and schizophrenia (Alemany-Navarro *et al*. 2023), and higher risk of SARS-CoV-2 infection in the general population. The lack of significant association in our cohort might be explained by several factors. First, at the time of this study the population incidence of confirmed COVID-19 infections in Australia was low compared with many other countries. Australia was almost free of COVID-19 until early 2022 (World Health Organization (WHO), 2023), when over 93% of the population older than 16 years old had been fully vaccinated (Australian Government, 2022). Therefore, the vast majority of AGDS participants who became infected were vaccinated unlike participants from the previously mentioned studies, who became infected when unvaccinated. Given that vaccines significantly reduce the risk of infection and reinfection (Flacco et al., 2022; Zheng et al., 2022), our results might be influenced by a vaccination effect. Second, unlike prior studies, our study was conducted in a cohort of people with a history of depression. Current evidence is inconclusive regarding whether people with depression have an increased risk of SARS-CoV-2 infection compared to people without depression (Bertolini et al., 2023) with some studies suggesting that commonly used antidepressants such as fluoxetine or sertraline could prevent viral infection by SARS-CoV-2 (Y. Chen et al., 2022b; Clelland, Ramiah, Steinberg, & Clelland, 2021; Fred et al., 2022). Therefore, the potential increased use of antidepressants in our cohort might act as a confounding factor, as antidepressants might reduce the susceptibility to SARS-CoV-2 infection, thus masking the effect of the psychiatric genetic risk scores on infection rates. Finally, when restricting our sample to individuals with depression, we are capturing particularly those individuals with higher genetic risk for depression and other mental disorders than what population-based studies do. This phenomenon, known as Berkson's bias, could lead to an underestimation of the effect of the genetic risk for the tested mental disorders on SARS-CoV-2, potentially explaining the lack of association found in our results (Griffith et al., 2020; Lu, Gonsalves, & Westreich, 2024).

Prior studies of post-infective syndromes highlight that concurrent mood disorders, and other individual behavioral traits, predict ongoing ill-health (Cvejic et al., 2019). However, studies examining the link between the genetic risk for mental disorders (as distinct from phenotypic expressions) and long COVID, and other post-infective syndromes, are lacking. Nevertheless, epidemiological studies have reported an increased risk of long COVID among people with depression and other mental disorders, which could potentially be caused by the pro-inflammatory environment present in some mental disorders (Reme, Gjesvik, & Magnusson, 2023; Wang et al., 2022). This suggests that genetic factors associated with these disorders might be contributing to the increased risk of long COVID. However, we did not find a significant association between the genetic predisposition to depression, bipolar disorder, anxiety, and schizophrenia and a higher risk of developing long COVID, suggesting that the increased risk of long COVID reported in people with depression might not be driven by genetic factors associated with these disorders. Nevertheless, factors such as vaccination, which has been reported to reduce the risk of long COVID (Richard et al., 2023), might be influencing our results. Further studies involving larger and diverse cohorts, and accounting for vaccination status and use of psychiatric medications are needed to better understand the complex interplay between genetics, mental disorders, and SARS-CoV-2 infection and long COVID-19.

We explored whether a higher genetic risk for the selected mental disorders predicted greater levels of COVID-related stress and COVID-19 burnout. We found that a higher PRS for depression was linked to higher levels of COVID-related stress, although this association did not withstand multiple testing correction. However, the depression PRS significantly predicted higher COVID-19 burnout. A higher PRS for bipolar disorder predicted lower COVID-19 burnout, but only at a nominal level. In sex-stratified analysis these associations were maintained in women (although only nominally), while no significant results were obtained in men. Nevertheless, the lack of significant results in men might be explained by a reduced sample size, which was three times smaller than that of women. We hypothesized that anxiety symptoms might influence the identified association between genetic predisposition to depression and higher levels of COVID-19 burnout. Results from the mediation analysis showed that anxiety symptoms, conducted in a subset of the sample, explained a substantial portion of the association between genetic predisposition to depression and COVID-19 burnout (78%), to the extent that the direct effect of the genetic factors disappeared. This result suggests that the higher risk of COVID-19 burnout reported in people with depression is predominantly driven by anxiety symptoms. COVID-19 burnout can have a serious impact on both mental and physical wellbeing, affecting the individual's ability to function efficiently (World Health Organization. Regional Office for Europe, 2020). In addition, current evidence suggests that burnout can result in reluctance to adhere to anti-pandemic measures (Lilleholt, Zettler, Betsch, & Böhm, 2023). Hence, from a population-health perspective, the much wider promotion of specific cognitive or behavioral interventions that target anxiety symptoms (and that can be self-administered or facilitated by digital technologies) (Linardon et al., 2024) early during a pandemic, or at other times of spikes in community-acquired viral infections, may well deliver significant mental health benefits. Such interventions focus on reduction in prolonged arousal, challenging irrational thoughts or fears and maintenance of regular 24 h sleep–wake cycles. Most notably, those positive effects are largely likely to be derived in people with pre-existing depression, regardless of their genetic risk for the disorder.

Finally, we hypothesized that resilience could moderate the identified associations between the PRS for depression and COVID-19 burnout. However, although higher resilience predicted lower COVID-19 burnout, it did not moderate the association between genetic risk for depression and COVID-19 burnout. One potential reason for the absence of a moderating effect may be the relatively low levels of resilience within our population (mean BRS = 2.8, s.d. = 0.8) (Chmitorz et al., 2018; Soer et al., 2019), which could result in insufficient variation to detect a moderating effect in our analysis. Additionally, although resilience is a known protective factor for mental health, available evidence has not identified a moderation effect of resilience on the association between the genetic load for depression and the manifestation of depression (Navrady, Adams, Chan, Ritchie, & Mcintosh, 2018).

The results of our study should be considered in the context of some limitations. First, AGDS participants were predominantly women of European ancestry, so our findings may not be generalizable to other populations and studies. Second, our sample was significantly older and had a lower PRS for schizophrenia compared to those lost to follow-up. This may bias our findings toward older individuals and those with a lower genetic predisposition for schizophrenia, potentially limiting the generalizability of our results. Third, SARS-CoV-2 infection and long COVID were self-reported rather than clinically diagnosed. Nevertheless, self-reported SARS-CoV-2 infection and symptoms have been shown to be reliable indicators of SARS-CoV-2 infection (Adorni et al., 2020; McCarthy et al., 2022). Fourth, while evidence suggests a protective effect of antidepressants against SARS-CoV-2 infection (Fred et al., 2022; Lee et al., 2022), we lacked data on current antidepressant use in our sample. Therefore, antidepressants could confound the association between genetic risk for mental disorders and infection. Fifth, our sample size for individuals with two SARS-CoV-2 infections was limited (*n* = 29), which may have reduced our power to detect an association. Sixth, we focused on the association of genetic risk and our outcomes, and we did not consider specific genotype–environment interactions.

In conclusion, we found no evidence that genetic risk for depression, bipolar disorder, schizophrenia, or anxiety predicted susceptibility to SARS-CoV-2 infection and long COVID-19 in people with history of depression. However, these results could be influenced by the unique conditions of the pandemic in Australia. A greater genetic load for depression predicted higher COVID-19 burnout; this association was fully mediated by anxiety symptoms, with no moderating effect from resilience. Therefore, ongoing and future pandemic interventions should focus on reducing anxiety symptoms to effectively support people with depression, regardless of their genetic susceptibility.

## Supporting information

Monistrol-Mula et al. supplementary material Monistrol-Mula et al. supplementary material

## Supplementary material

The supplementary material for this article can be found at https://doi.org/10.1017/S0033291724001983.
