# Supplementary material for: The effect of polygenic liability to mental disorders on COVID-19 outcomes in people with depression: the mediating role of anxiety
Source: Psychol Med. 2024 Nov 18;54(15):4140–9. doi: 10.1017/S0033291724001983 (PMC11650167; doi:10.1017/S0033291724001983)
Supplement: Monistrol-Mula et al. supplementary material [file S0033291724001983sup001.docx]

SUPPLEMENTARY MATERIAL

Annex 1. Results of regression analysis for the clinical and psychological COVID-19 outcomes including all PRSs in the same model (N = 4,405)

| Outcome | | PRS depression | | PRS bipolar disorder | | PRS schizophrenia | | PRS anxiety | |  |
| --- | --- | --- | --- | --- | --- | --- | --- | --- | --- | --- |
|  |  | **RRR (95% CI)** | ***p*** | **RRR (95% CI)** | ***p*** | **RRR (95% CI)** | ***p*** | **RRR (95% CI)** | ***p*** |  |
| SARS-CoV-2 infection | Never | Ref |  | Ref |  | Ref |  | Ref |  |  |
|  | Once | 1.02  (0.93-1.11) | 0.694 | 0.94  (0.88-1.03) | 0.187 | 1.02  (0.94-1.12) | 0.514 | 1.05  (0.97-1.14) | 0.237 |  |
|  | Twice | 1.19  (0.77-1.84) | 0.438 | 1.14  (0.78-1.67) | 0.512 | 0.88  (0.57-1.35) | 0.548 | 1.19  (0.79-1.80) | 0.397 |  |
|  |  | **OR (95% CI)** | ***p*** | **OR (95% CI)** | ***p*** | **OR (95% CI)** | ***p*** | **OR (95% CI)** | ***p*** |  |
| Long COVID | No | Ref |  | Ref |  | Ref |  | Ref |  |  |
|  | Yes | 0.96  (0.80-1.16) | 0.671 | 1.06  (0.89-1.25) | 0.524 | 0.96  (0.80-1.16) | 0.693 | 1.04  (0.88-1.24) | 0.637 |  |
| Outcome |  | **β (SE)** | ***p*** | **β (SE)** | ***p*** | **β (SE)** | ***p*** | **β (SE)** | ***p*** | **Adj R2** |
| COVID-related stress |  | 0.20 (0.08) | 0.017 | -0.07 (0.07) | 0.378 | -0.14 (0.09) | 0.091 | -0.02 (0.08) | 0.757 | 0.024 |
| COVID-19 burnout |  | 0.39 (0.13) | **0.004** | -0.24 (0.12) | 0.048 | -0.23 (0.13) | 0.085 | 0.01 (0.13) | 0.94 | 0.090 |

Relative Risk Ratios (RRR) with 95% CI and P-values are displayed for categorical variables. Odd ratios (OR) with 95% confidence intervals (95% CI) and P-values are displayed for bivariate variables. Estimates (β) with standard errors (SE) and P-values are displayed for continuous variables. Adjusted R2 are displayed for linear models. Analysis were made separately for each outcome and adjusted for age, sex, severity of depression and 8 genetic ancestry principal components. Bold font shows significant associations (corrected significance threshold p<0.013)

Annex 2. Results of regression analysis for the clinical and psychological COVID-19 outcomes adjusting for all covariates (N = 4,405)

| Outcome | | PRS depression | | | PRS bipolar disorder | | | PRS schizophrenia | | | PRS anxiety | | |
| --- | --- | --- | --- | --- | --- | --- | --- | --- | --- | --- | --- | --- | --- |
|  |  | **RRR (95% CI)** | ***p*** | | **RRR (95% CI)** | ***p*** | | **RRR (95% CI)** | ***p*** | | **RRR (95% CI)** | ***p*** | |
| SARS-CoV-2 infection | Never | Ref |  | | Ref |  | | Ref |  | | Ref |  | |
|  | Once | 1.03  (0.95-1.13) | 0.399 | | 0.96  (0.88-1.04) | 0.286 | | 1.01  (0.93-1.11) | 0.730 | | 1.06  (0.99-1.15) | 0.112 | |
|  | Twice | 1.21  (0.80-1.84) | 0.357 | | 1.12  (0.77-1.65) | 0.546 | | 0.90  (0.59-1.38) | 0.626 | | 1.27  (0.86-1.87) | 0.223 | |
|  |  | **OR (95% CI)** | ***p*** | | **OR (95% CI)** | ***p*** | | **OR (95% CI)** | ***p*** | | **OR (95% CI)** | ***p*** | |
| Long COVID | No | Ref |  | | Ref |  | | Ref |  | | Ref |  | |
|  | Yes | 0.96  (0.80-1.14) | 0.641 | | 1.09  (0.92-1.29) | 0.316 | | 0.96  (0.79-1.16) | 0.653 | | 1.04  (0.88-1.23) | 0.669 | |
| Outcomes |  | **β (SE)** | ***p*** | **adj R^2^** | **β (SE)** | ***p*** | **adj R^2^** | **β (SE)** | ***p*** | **adj R^2^** | **β (SE)** | ***p*** | **adj R^2^** |
| COVID-related stress |  | 0.18  (0.08) | 0.023 | 0.03 | -0.06  (0.08) | 0.462 | 0.03 | -0.16  (0.09) | 0.054 | 0.03 | 0.02  (0.08) | 0.840 | 0.03 |
| COVID-19 burnout |  | 0.33  (0.12) | **0.009** | 0.10 | -0.19  (0.12) | 0.09 | 0.10 | -0.26  (0.14) | 0.062 | 0.10 | 0.05  (0.12) | 0.694 | 0.10 |

Relative Risk Ratios (RRR) with 95% CI and P-values are displayed for categorical variables. Odd ratios (OR) with 95% confidence intervals (95% CI) and P-values are displayed for bivariate variables. Estimates (β) with standard errors (SE) and P-values are displayed for continuous variables. Adjusted R2 are displayed for linear models. Analysis were made separately for each outcome and adjusted for age, sex, severity of depression, number of physical diseases, mental comorbidities, smoking, and 8 genetic ancestry principal components. Bold font shows significant associations (corrected significance threshold p<0.013)

Annex 3. Results of sex-stratified regression analysis for the clinical and psychological COVID-19 outcomes

| Females (n = 3,366) | | | | | | | | | | | | | |
| --- | --- | --- | --- | --- | --- | --- | --- | --- | --- | --- | --- | --- | --- |
| Outcome | | **PRS depression** | | | **PRS bipolar disorder** | | | **PRS schizophrenia** | | | **PRS anxiety** | | |
|  |  | **RRR (95% CI)** | ***p*** | | **RRR (95% CI)** | ***p*** | | **RRR (95% CI)** | ***p*** | | **RRR (95% CI)** | ***p*** | |
| SARS-CoV-2 infection | Never | Ref |  | | Ref |  | | Ref |  | | Ref |  | |
|  | Once | 1.03  (0.93-1.13) | 0.600 | | 0.93  (0.85-1.01) | 0.103 | | 1.03  (0.94-1.13) | 0.540 | | 1.07  (0.98-1.17) | 0.144 | |
|  | Twice | 1.10  (0.67-1.80) | 0.703 | | 1.09  (0.72-1.66) | 0.680 | | 0.94  (0.59-1.50) | 0.784 | | 1.43  (0.90-2.25) | 0.128 | |
|  |  | **OR (95% CI)** | ***p*** | | **OR (95% CI)** | ***p*** | | **OR (95% CI)** | ***p*** | | **OR (95% CI)** | ***p*** | |
| Long COVID | No | Ref |  | | Ref |  | | Ref |  | | Ref |  | |
|  | Yes | 0.99  (0.81-1.22) | 0.913 | | 1.04  (0.87-1.25) | 0.653 | | 1.00  (0.82-1.22) | 0.994 | | 1.00  (0.83-1.21) | 0.993 | |
| Outcomes |  | **β (SE)** | ***p*** | **adj R^2^** | **β (SE)** | ***p*** | **adj R^2^** | **β (SE)** | ***p*** | **adj R^2^** | **β (SE)** | ***p*** | **adj R^2^** |
| COVID-related stress |  | 0.23  (0.10) | 0.02 | 0.025 | -0.06  (0.09) | 0.509 | 0.025 | -0.18  (0.10) | 0.059 | 0.025 | 0.022  (0.09) | 0.811 | 0.025 |
| COVID-19 burnout |  | 0.36  (0.15) | 0.019 | 0.093 | -0.30  (0.14) | 0.028 | 0.093 | -0.28  (0.15) | 0.062 | 0.093 | 0.13  (0.14) | 0.403 | 0.093 |
| Males (n = 1,051) | | | | | | | | | | | | | |
| Outcome | | **PRS depression** | | | **PRS bipolar disorder** | | | **PRS schizophrenia** | | | **PRS anxiety** | | |
|  |  | **RRR (95% CI)** | ***p*** | | **RRR (95% CI)** | ***p*** | | **RRR (95% CI)** | ***p*** | | **RRR (95% CI)** | ***p*** | |
| SARS-CoV-2 infection | Never | Ref |  | | Ref |  | | Ref |  | | Ref |  | |
|  | Once | 0.99  (0.83-1.19) | 0.930 | | 1.03  (0.87-1.23) | 0.708 | | 0.98  (0.81-1.20) | 0.880 | | 0.96  (0.81-1.15) | 0.674 | |
|  | Twice | 1.24  (0.47-3.27) | 0.663 | | 1.42  (0.54-3.75) | 0.476 | | 0.53  (0.17-1.63) | 0.267 | | 0.72  (0.27-1.92) | 0.514 | |
|  |  | **OR (95% CI)** | ***p*** | | **OR (95% CI)** | ***p*** | | **OR (95% CI)** | ***p*** | | **OR (95% CI)** | ***p*** | |
| Long COVID | No | Ref |  | | Ref |  | | Ref |  | | Ref |  | |
|  | Yes | 0.85  (0.55-1.30) | 0.448 | | 1.16  (0.77-1.75) | 0.514 | | 0.77  (0.47-1.27) | 0.278 | | 1.23  (0.82-1.86) | 0.247 | |
| Outcomes |  | **β (SE)** | ***p*** | **adj R^2^** | **β (SE)** | ***p*** | **adj R^2^** | **β (SE)** | ***p*** | **adj R^2^** | **β (SE)** | ***p*** | **adj R^2^** |
| COVID-related stress |  | 0.11  (0.16) | 0.520 | 0.010 | -0.08  (0.15) | 0.588 | 0.010 | 0.04  (0.18) | 0.822 | 0.010 | -0.15  (0.16) | 0.342 | 0.010 |
| COVID-19 burnout |  | 0.49  (0.28) | 0.079 | 0.047 | 0.03  (0.26) | 0.914 | 0.047 | -0.08  (0.30) | 0.783 | 0.047 | -0.34  (0.26) | 0.203 | 0.047 |

Relative Risk Ratios (RRR) with 95% CI and P-values are displayed for categorical variables. Odd ratios (OR) with 95% confidence intervals (95% CI) and P-values are displayed for bivariate variables. Estimates (β) with standard errors (SE) and P-values are displayed for continuous variables. Adjusted R2 are displayed for linear models. Analysis were made separately for each outcome and PRS, and adjusted for age, sex, severity of depression and 8 genetic ancestry principal components. Bold p-values show significant associations (corrected significance threshold p<0.013).

Annex 4. Moderation analysis for resilience on the association between PRS for depression and COVID-related stress and COVID-19 burnout, and PRS for bipolar and COVID-19 burnout (n = 3,326)

|  |  |  | Covid-19 burnout | |
| --- | --- | --- | --- | --- |
|  |  |  | **β (SE)** | ***p*** |
| PRS depression | Model 1 | PRS depression | 0.27 (0.14) | 0.052 |
|  | Model 2 | Resilience | -2.76 (0.16) | <0.001 |
|  | Model 3 | PRS depression | 0.23 (0.14) | 0.092 |
|  |  | Resilience | -2.77 (0.17) | <0.001 |
|  |  | PRS depression*resilience | 0.12 (0.17) | 0.492 |

Model 1 included only resilience and the covariates; model 2 included the PRS and the covariates; model 3 included resilience, the PRS, the PRS*resilience interaction, and the covariates. Analyses were adjusted for age, sex, severity of depression and 8 principal components.
